# Chiral MoS_2_ Quantum Dots: Dual‐Mode Detection Approaches for Avian Influenza Viruses

**DOI:** 10.1002/gch2.201700071

**Published:** 2018-03-12

**Authors:** Syed Rahin Ahmed, Suresh Neethirajan

**Affiliations:** ^1^ BioNano Laboratory School of Engineering University of Guelph Guelph Ontario N1G 2W1 Canada

**Keywords:** avian influenza virus, magnetofluorescent nanohybrids, MoS_2_ QDs, nanosensors

## Abstract

Molybdenum disulfide (MoS_2_), a type of transition metal dichalcogenide material, has emerged as an important class among 2D systems. When 2D MoS_2_ materials are reduced to 0D quantum dots (QDs), they introduce new optical properties that point to several potential technological advantages in electronic, magnetic, optical, and catalytic properties. In this study, a simple way to produce chiral MoS_2_ QDs from MoS_2_ nanopowder is presented using l(+)‐ascorbic acid as a reducing agent. The calculated quantum yield of QDs is 11.06%. Experimental results reveal that the size of QDs is uniformly monodispersed (2–3 nm) and have a blue emissive fluorescence peak and circular dichroism (CD) peak located at 420 and 330 nm, respectively. Furthermore, a dual‐mode detection system based on fluorescence and chirality is performed using as‐synthesized MoS_2_ QDs, where QDs are conjugated with anti‐hemagglutinin antibodies of avian influenza virus and made into an immunobridge in the presence of target virus and anti‐neuraminidase antibodies conjugated magnetic nanoparticles (MNPs). The photoluminescence and CD spectra of unconjugated QDs after separated magnetochirofluorescent (MNPs‐QDs) nanohybrids by external magnets enables influenza virus A (H5N1) detection with the limit of detection value of 7.35 and 80.92 pg mL^−1^, respectively.

## 1 Introduction

Nanoscale semiconductor materials possess various emergent properties that have potential technological significance in numerous applications, such as biosensors, bioimaging, light‐emitting devices, memory devices, and photovoltaic devices. For these reasons, significant effort has been given to introducing new semiconductor materials with numerous innovative properties and applications.^1^, ^2^, ^3^, ^4^, ^5^, ^6^ In particular, the discovery of 2D materials, that is, graphene, has brought more attention to other layered materials due to their intrinsic electronic properties and high specific surface area.^7^, ^8^, ^9^

Among 2D materials, molybdenum disulfide (MoS_2_) belongs to the transition metal dichalcogenide (TMD) class and has received great amount of interest due to its inherently different physical properties, most importantly, the existence of intrinsic tunable bandgaps. MoS_2_ shows layer thickness‐dependent tunable bandgap; for example, from indirect bandgap (1.29 eV for bulk form) to direct bandgap (1.9 eV for monolayer).^10^, ^11^ Thus, the arrangement of atoms in MoS_2_ enables the preparation of more active sites with a higher surface‐to‐volume ratio than bulk MoS_2_ counterparts, and introduces great potential advances in the field of electronics, optics, and catalytics. Besides the effect of layer thickness, the nanoscale dimensions of MoS_2_ revealed a new class of semiconductor materials (known as MoS_2_ QDs) due to quantum confinement and edge effects, opening a new horizon of unprecedented scientific and technological applications.^12^

In general, the synthetic procedures of MoS_2_ QDs can be divided into bottom‐up and top‐down processes. In the bottom‐up process, MoS_2_ QDs can be synthesized through a hydrothermal process. For example, Gu et al. proposed ethanol‐thermal synthesis of bright blue MoS_2_QDs, with an average size of 2.9 nm. The overall time to derive the final product was about 20 h.^13^ A bottom‐up synthesis method of water‐soluble MoS_2_ QDs was presented by the same authors using poly(tetrafluoroethylene) (Teflon) autoclave at 200 °C for 8 h.^14^ Wang and Ni reported another hydrothermal way to derive MoS_2_ QDs, using Na_2_MoO_4_·2H_2_O and l‐cysteine at 200 °C for 36 h.^15^ All of these processes were either time consuming or required several steps. In the top‐down process, MoS_2_ QDs were synthesized through mechanical or chemical exfoliation techniques. For example, Ha et al. presented a laborious, Li‐intercalation method to synthesize blue‐emitting MoS_2_ QDs.^16^ Li et al. prepared MoS_2_ QDs through an electrochemically induced Fenton (electro‐Fenton) reaction.^17^ Although this top‐down process enabled the preparation of MoS_2_QDs, some disadvantages, that is, size distribution and nature of edges limit their applications such as development of sensitive biosensors.

Recently, chiral metamaterials have gained substantial research interest, perhaps due to an enhanced optical response in comparison to the natural chiral molecules. The chiroptical response of chiral metamaterials is several orders of magnitude higher than that of chiral biomolecules. Meanwhile, advancement of novel nanofabrication techniques has also enabled the creation of chiral nanostructures which exhibit not only a high amount of circular dichroism (CD) and optical rotatory dispersion, but also extensively enhances the signals obtained from target molecules, which in principle provides a sufficient method to detect with a very high sensitivity.^18^, ^19^, ^20^, ^21^, ^22^, ^23^, ^24^, ^25^, ^26^, ^27^, ^28^, ^29^, ^30^, ^31^ In particular, optical chirality integrated with fluorescence in a nanosystem represents an attractive area of research and has received a great deal of attention because of its potential for optically active components in devices and its ability to offer a wide range of research and development possibilities in diverse areas of physics, chemistry, and life sciences.^32^, ^33^, ^34^, ^35^ Chiral fluorescent nanomaterials may exhibit strong coupling with incident photons and can potentially lead to unusual optical responses that are essential for chiral and fluorescence sensing as well as other optical devices. Chiral fluorescent MoS_2_ QDs are still elusive, even though little effort has been given to prepare bare MoS_2_ QDs. Considering these factors, it is crucial to investigate and introduce chiral MoS_2_ QDs for the development of novel devices.

In this study, we have designed a bottom‐up, time‐effective, and easy way to prepare ultrasmall uniform chiral MoS_2_ QDs with blue fluorescence emission properties. Simply, l(+)‐ascorbic acid and MoS_2_ nanopowder were mixed in aqueous media and kept in an autoclave for 1 h at 120 °C. The initial dark gray colored solution then turned yellow, indicating the formation of MoS_2_QDs. As‐prepared MoS_2_ QDs showed bright blue color under UV light. Synthesized QDs exhibited chirality and high dispersibility in aqueous media and were smaller in size; hence, they can be employed for detecting avian influenza virus proteins and virus cultures from complex blood samples.

We have chosen influenza virus detection as a model analyte because of its high pathogenicity in the poultry industry as well as the associated concerns to humans due to its transmission ability from infected domestic poultry to humans and then from person‐to‐person. The high mortality rate of the avian influenza virus in both poultry and humans could have a significant effect on the global economy, with a particularly strong impact on social health care.^36^, ^37^, ^38^, ^39^ Therefore, a rapid and sensitive bioassay for influenza virus detection could be particularly useful for the timely response to overcome future pandemic threats. The practical applicability of the proposed dual‐mode sensor was demonstrated using an avian influenza A (H4N6) virus culture sample in this study.

## 2 Results and Discussion

**Scheme 1:**
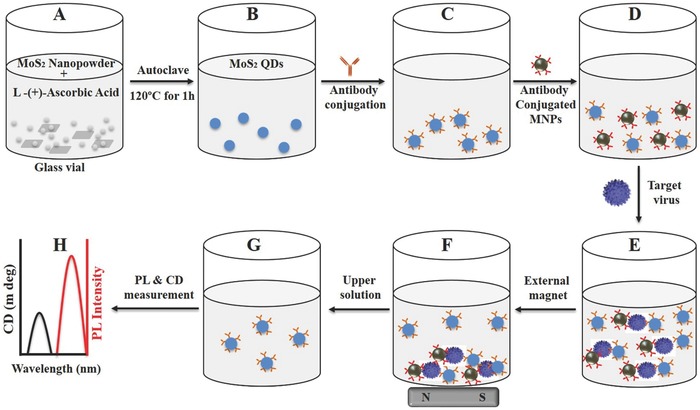
Schematic presentation of the overall experimental design: A) MoS_2_ nanopowder and ascorbic acid are mixed in a glass vial; B) Upon autoclaving, MoS_2_ QDs were formed; C) QDs were conjugated with antibodies through a layer‐by‐layer (LBL) method; D) antibody‐conjugated MNPs were added to it; E) target virus was added and nanostructured magnetochirofluorescent was formed, F) which were separated by an external magnet; G) upper solution was collected and H) tested for optical responses.

Over the last few years, nanohybrid materials have gained significant research interest, and various approaches to conjugate different materials in one nanostructure have been described, either in a direct synthesis manner or by the conjugation of separate nanomaterials. In particular, magnetochirofluorescent nanohybrids are favorable materials for diverse applications in biomedical field, that is, fluorescence sensing, fluorescence imaging, magnetic resonance imaging, and magnetic separation of target analytes.^40^, ^41^ In this study, we applied a two‐step process to make magnetochirofluorescent nanohybrids through immunoreaction for avian influenza virus detection. At first, nanocrystals of 2D materials (MoS_2_ QDs) and MNPs were synthesized, conjugated with antitarget analyte antibodies, and mixed in a microtube (see **Scheme** **1**). At this stage, no interaction exists between bioconjugated MoS_2_ QDs and MNPs, and they will stay apart from each other. Antibody‐conjugated MoS_2_ QDs and MNPs will then come closer together, forming a magnetochirofluorescent nanohybrid structure through immunoreaction with the addition of target analyte. The resulting nanostructured magnetochirofluorescent can be separated from the unconjugated part using an external magnetic field, while the photoluminescence (PL) intensity and CD of the remaining unconjugated MoS_2_ QDs can tell us about the analytes' concentration (Scheme 1). Here, both the PL intensity and CD response will decrease with an increasing target virus concentration, since most of the QDs will participate in magnetochirofluorescent hybrid construction and will be separated by external magnetic force. By varying the analytes' concentration, this study attempts to establish chiral MoS_2_ QD‐based fluorescence and chiral biosensor development for the avian influenza virus.

### 2.1 Characterization of MOS_2_‐QDs and MNPs

**Figure 1:**
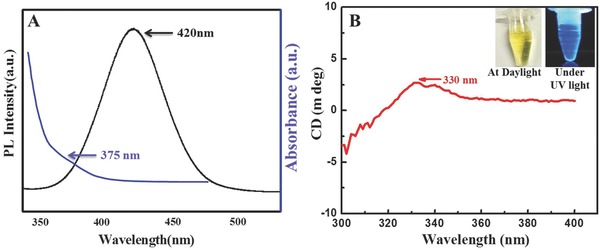
Optical properties of the synthesized MoS_2_ QDs: A) fluorescence and UV–vis spectra of MoS_2_ QDs; B) circular dichroism spectra of circular dichroism (inset: color of solution under daylight and UV light).

The photoluminescence intensity and UV–vis spectrum of MoS_2_ QDs were located at 420 nm and 375 nm, respectively, as shown in **Figure** **1**A. As‐prepared MoS_2_ QDs showed chirality upon circular dichroism measurement located at 332 nm (Figure 1B). Synthesized QDs exhibited a yellow color by the naked eye, whereas under UV light, a strong blue color was apparent (Figure 1B inset).

The quantum yield (QY) of synthesized MoS_2_ QDs was determined using Rhodamine 6G dissolved in ethanol as a reference standard (Φ_F_ = 0.95). The following Equation (1) was used to calculate QY

(1) $$\Phi_{F}=\Phi_{F\left(\mathrm{Std}\right)}\frac{F.\;\;A_{\mathrm{Std}\;}.\;\;n^{2}}{F_{\mathrm{Std}\;\;}.\;\;A_{}.\;n_{\mathrm{Std}}^{2}}$$

In the above equation, Φ_F_ represents the PL QY of the MoS_2_ QDs, Φ_F(Std)_ represents the PL of the standard, *F* and *F*_Std_ denote the integrated sums of the PL intensity of the MoS_2_ QDs and standard respectively, *A* and *A*_Std_ signify the optical densities of the MoS_2_ QDs and the standard respectively, and *n* ^2^ and *n* ^2^_Std_ represent the refractive indexes of the solvents used to dissolve the QDs and the standard, respectively. The corresponding PL QY value of the MoS_2_ QDs was 11.06%.

The fluorescence decay dynamics of excited states of nanocrystals is significant in the understanding of its electro‐optic and catalytic properties. Here, the fluorescence lifetime of MoS_2_ QDs was analyzed using Horiba time‐resolved fluorimeter (Model PPD‐650, Ontario, Canada). Samples were excited at 336 nm using a Delta Diode laser, and the fluorescence intensity was measured as a function of time at the wavelength of maximum emission.

The fluorescence decays were then analyzed by fitting to a sum of exponentials according to the following Equation (2), 42

(2) $$I(t)=I_{0}(t)\underset{i}{\Sigma }\alpha_{i}\exp\left[\frac{-t}{\tau_{i}}\right]$$

**Figure 2:**
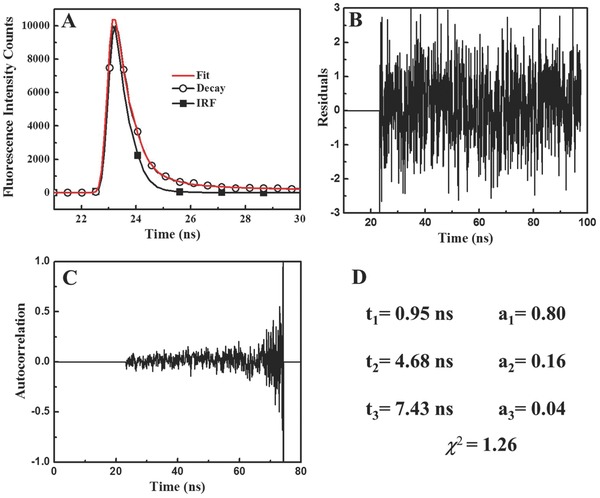
Fluorescence lifetime of MoS_2_: A) fluorescence decays profile of MoS_2_QDs, where (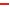) fit, (○) decay, and (■) instrument response function; B) the residuals, and C) autocorrelation of the residuals; and D) obtained three lifetimes and χ^2^ value.

In Equation (2), *I*_0_(*t*) represents the fluorescence intensity at *t* = 0, while α and *τ* denote the pre‐exponential factor and lifetime in time bin *i*, respectively. The lifetime decay profile of MoS_2_ QDs is shown in **Figure** **2**A. The residuals as well as their autocorrelation were evenly distributed around zero (Figure 2B,C). Three lifetimes were acquired for a good fit, characterized by an χ^2^ value (Figure 2D). The average lifetime of the quantum dots was calculated according to Equation (3) below, and the calculated QD lifetime equaled 4.76 ns

(3) $$\left\langle \tau \right\rangle =\frac{\Sigma \alpha_{i}\tau_{i}}{\Sigma \alpha_{i}}$$

**Figure 3:**
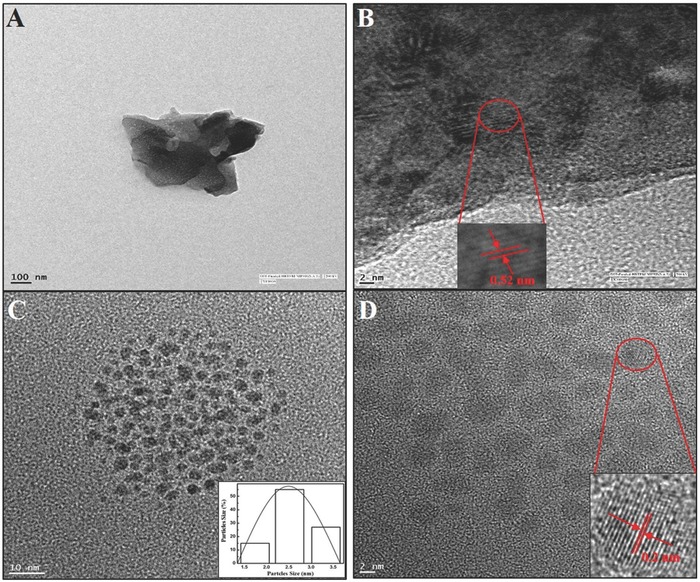
Microscopic study of the MoS_2_QDs and nanopowder: A) HRTEM image of MoS_2_ QDs and nanopowder; B) lattice pattern of MoS_2_ nanopowder; and C) HRTEM image of well‐dispersed MoS_2_ QDs (inset: size distribution profile of QDs) and D) the lattice pattern of MoS_2_ QDs.

High‐resolution transmission electron images (HRTEMs) of bulk MoS_2_ nanomaterials and MoS_2_ QDs were acquired on an FEI Titan 8‐300 TEM, equipped with a CEOS hexapole image corrector. The microscope was operated at 300 kV in bright‐field TEM mode. We then dispersed 20 μL of QDs on a Ted Pella ultrathin carbon support film grid and air‐dried. The sample was plasma‐cleaned in a GatanSolarus plasma cleaner using an Ne, H, and Ar mixture for 30 s at 30 W. As shown in **Figure** **3**A, the length and diameter of bulk MoS_2_ nanopowder were a few hundred nanometers. As can be observed in Figure 3B, bulk MoS_2_ have parallel crystal fringes with an interlayer distance of 0.60 nm, corresponding to the interplanar spacing of the MoS_2_ (002) plane. The HRTEM image of as‐prepared MoS_2_QDs demonstrated that QDs are well dispersed and uniform in size, with an average size of 2.5 nm (Figure 3C). Such well‐dispersed nanocrystals are considered advantageous for optical‐based sensor development, as they help to avoid aggregation and quenching issues. The closed HRTEM image confirmed that as‐prepared MoS_2_ QDs feature a highly paralleled and ordered lattice fringe structure and interlayer spacing of about 0.20 nm, as indicated by the (006) lattice plane of the hexagonal crystal MoS_2_ (Figure 3D).

**Figure 4:**
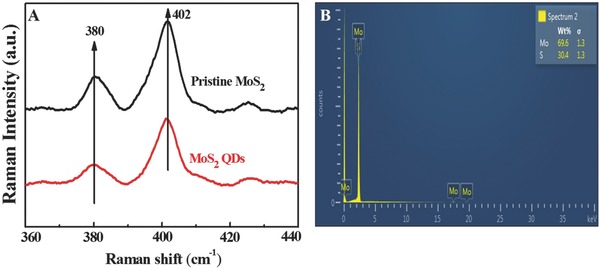
Raman spectra and elementary analysis of MoS_2_ QDs: A) Raman spectra of pristine MoS_2_ nanopowder and MoS_2_ QDs; and B) EDX profile of MoS_2_ QDs.

Raman spectra of MoS_2_ QDs are shown in **Figure** **4**A. Experimental results of Raman spectra revealed two phonon modes ($E_{2g}^{1}$ and A_1g_) located at 380 and 402 cm^−1^, respectively, for both MoS_2_ pristine and MoS_2_ QDs, where $E_{2g}^{1}$ mode is associated with the opposite vibration of two sulfur atoms with respect to the molybdenum atom, and A_1g_ mode is related to the out‐of‐plane vibration of sulfur atoms in the opposite direction.^43^ An energy‐dispersive X‐ray (EDX) analysis was also performed to determine the elemental composition of as‐prepared MoS_2_ QDs (Figure 4B). The presence of Mo and S atoms was confirmed in MoS_2_ QDs through characteristic peak positions. The atomic ratio of S to Mo was calculated at about 2.28, which is very close to the theoretical value of MoS_2_.

**Figure 5:**
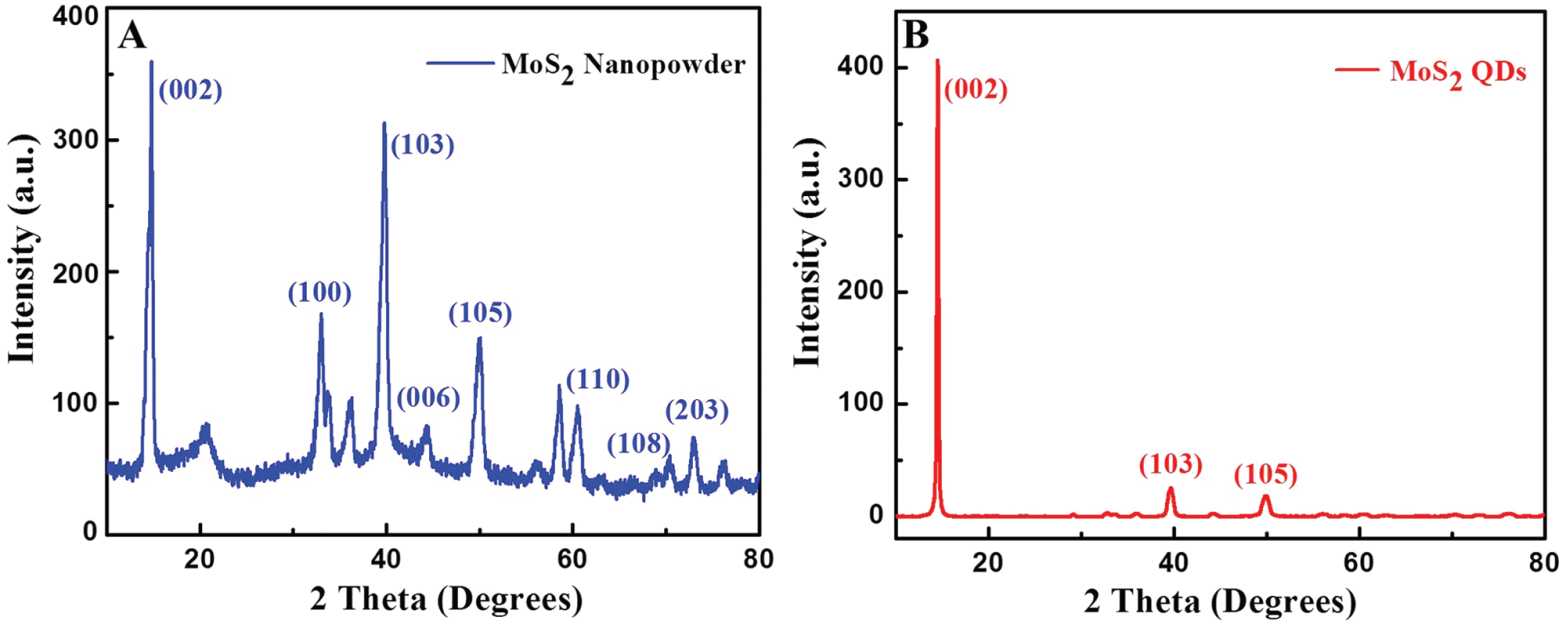
XRD patterns of A) MoS_2_ nanopwder and B) MoS_2_ QDs.

**Figure** **5**A showed a strong diffraction peak at 2θ = 14.42 and other peaks at 2θ = 32.84, 39.65, 44.81, 49.34, 60.53, 71.23, and 73.56 for bulk MoS_2_ corresponds to the (002), (100), (103), (006), (105), (110), (108), and (1203) planes, respectively. For the MoS_2_ QDs (Figure 5B), only three peaks can be detected at 2θ = 14.53 (002), 39.91 (103), and 51.09 (105), and other peaks disappeared, indicating the formation of mono‐ or few‐layered MoS_2_ QDs.^13^

**Figure 6:**
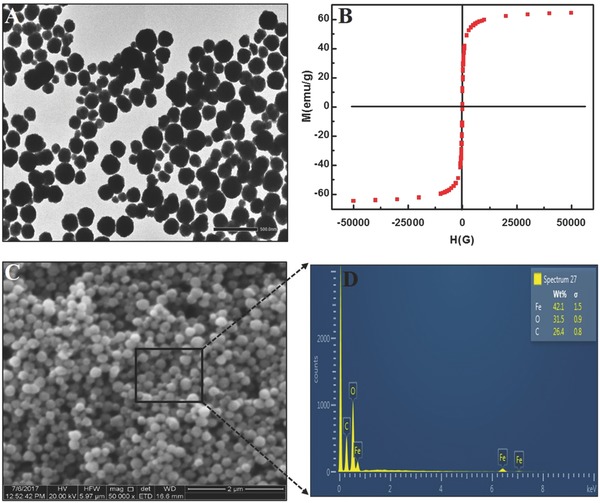
Characterization of the magnetic nanoparticles (MNPs): A) TEM image of MNPs; B) SQUID measurement of MNPs; C) SEM image of MNPs; and D) EDX profile of MNPs.

Besides MoS_2_QDs, the obtained TEM image of synthesized MNPs showed that magnetite particles are nearly spherical in shape at around 250 nm in size (**Figure** **6**A). The magnetic properties of as‐synthesized magnetite nanoparticles are shown in Figure 6B. The particles have no remanence or coercivity at 300 K, indicating that the particles respond magnetically to an external magnetic field and exhibit a superparamagnetic nature due to no hysteresis. MNPs possess a magnetic saturation value at 60.0 emu g^−1^, making them highly susceptible to external magnetic fields. These properties make MNPs a useful candidate as a separation tool from complex mixtures. A scanning electron microscopy (SEM) image of as‐synthesized MNPs is depicted in Figure 6C; a portion of it was used for elementary analysis. EDX results confirmed the presence of Fe and O atoms in MNPs, with a ratio of 1.33:1 (Figure 6D).

### 2.2 Confirmation of Antibodies Specificity toward Target Virus

The specificity of anti‐H5N1 HA (Ab 135382) and anti‐H5N1 NA antibodies for influenza virus A/Vietnam 1203/04/2009 (H5N1) was confirmed through enzyme‐linked immunosorbent assay (ELISA) method. As shown in Figure S1 (Supporting Information), a higher optical density was obtained for anti‐H5N1 HA (Ab 135382) and anti‐H5N1 NA antibodies in comparison to anti‐H5N2 HA and anti‐H7N9 HA antibodies, illustrating that anti‐H5N1 HA (Ab 135382) and anti‐H5N1 NA antibodies have a strong binding affinity toward influenza virus A/Vietnam 1203/04/2009 (H5N1).

### 2.3 Binding Confirmation of Anti‐H5N1 HA Ab 135382 with QDs and Anti‐H5N1 NA Ab with MNPs

The binding of anti‐H5N1 Ab 135382 and anti‐H5N1 NA Ab with nanomaterials was confirmed by ELISA. In Figure S2 (Supporting Information), a higher optical density was obtained for anti‐H5N1 Ab 135382, and anti‐H5N1 NA Ab confirmed the successful binding between antibodies and nanomaterials. A step‐by‐step variation in zeta potential measurements (Zetasizer Nano ZS, Malvern Instruments Ltd., Worcestershire, UK) also confirmed the successful conjugation of antibodies with QDs (Table S1, Supporting Information).

The amide bond formed between anti‐H5N1 NA antibodies and MNPs was further confirmed by an Fourier transform infrared (FTIR) spectrum. As shown in Figure S3 (Supporting Information), FTIR bands found at 3500–3700 and 1630–1690 cm^−1^ represents amide N—H stretching and amide C=O stretching, respectively.^40^, ^41^

**Figure 7:**
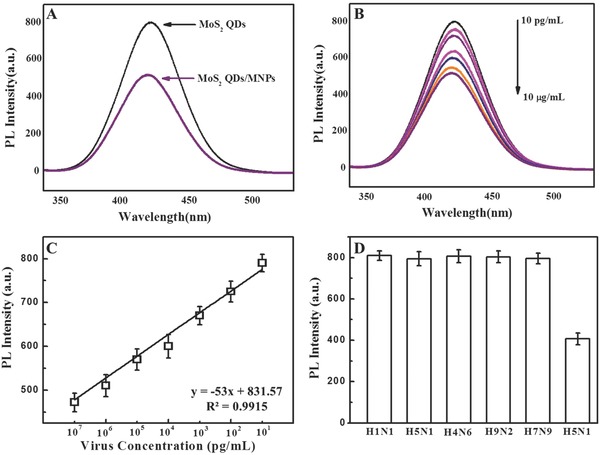
Fluorescence detection of avian influenza virus A (H5N1): A) PL response before and after nanohybrids formation; B) PL spectra of QDs with different concentrated target virus solution; C) calibration curve of PL intensity versus virus concentration; and D) selectivity results of fluorescence‐based sensor.

After confirming the binding antibodies with nanoparticles, an immunoassembly of magnetofluorescent was formed via the addition of various concentrated recombinant influenza virus A samples (H5N1) to antibody conjugated MoS_2_QDs and MNPs. Fluorescence intensity after separation of immune‐assembled magnetofluorescent nanohybrids decreased to ≈52% in comparison to the bare antibody conjugated MoS_2_ QDs with the addition of 10 μg mL^−1^ concentrated virus. The change in fluorescence intensity with different viral concentration was then carefully examined (**Figure** **7**A,B), and a calibration curve for fluorescence detection of avian influenza virus was constructed. The PL response with different concentrated influenza virus was observed in the range of 10 μg mL^−1^ to 10 pg mL^−1^ with an LOD value of 7.35 pg mL^−1^ (Figure 7C).

The selectivity test of the proposed fluorescent bioassay was implemented with other virus strains, namely, H1N1, H5N2, H7N8, and H7N9; a significant decrease in fluorescent response was obtained with the target avian influenza virus A (H5N1) in comparison to the other strains, demonstrating that the proposed fluorescent bioassay is selective only to the detection of target avian influenza virus A (H5N1) (Figure 7D). The sensitivity of the developed fluorescence sensor was validated with the conventional ELISA method and commercial colorimetric detection kit for avian influenza A (H5N1) (Table S2, Supporting Information). The visual color response of the commercial kit was up to 1 ng mL^−1^, indicating that the fluorescence response‐based bioassay is several orders of magnitudes sensitive than the conventional ELISA methods and commercial kits.

**Figure 8:**
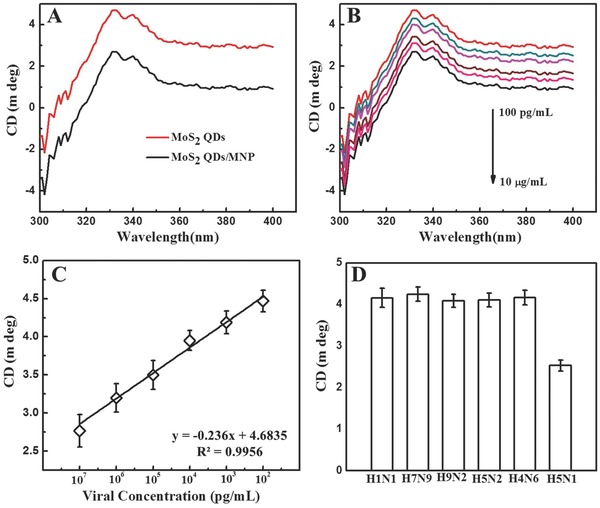
Chiral‐based detection of avian influenza virus A (H5N1): A) CD response before and after nanohybrids formation; B) CD spectra of QDs with different concentrated target virus; C) Calibration curve of CD intensity versus virus concentration; and D) selectivity results of chiral‐based sensor.

The chiral response of the supernatant solution after separation of magnetofluorescent nanohybrids was decreased to about 50% in comparison to the bare antibody conjugated MoS_2_QDs with an addition of 10 μg mL^−1^ avian influenza virus. Next, the changes of circular dichroism spectra with varying concentrated viral solutions were carefully examined (**Figure** **8**A,B), and a calibration curve for CD‐based detection of avian influenza virus was established. The CD responses with varying concentrated virus were observed to be in the range of 10 μg mL^−1^ to 100 pg mL^−1^, with an LOD value of 80.92 pg mL^−1^ (Figure 8C). Hence, the developed chiro‐biosensor is ten times more sensitive than the conventional ELISA methods and commercial kits (Table S2, Supporting Information). A series of different influenza viruses were also employed to check the selectivity of the proposed CD‐based sensor. As shown in Figure 8D, the developed sensor was highly selective only for the target analytes, that is, avian influenza virus A (H5N1).

We also explored the practical applicability of the developed fluorescence and chiral‐based biosensors for their ability to detect avian influenza A (H4N6) in the complex biological matrix such as chicken blood samples. Upon confirming the anti‐H4N6 antibody specificity toward virus culture samples, that is, avian influenza A (H4N6) virus (Figure S4, Supporting Information), and its binding confirmation with MoS_2_QDs and MNPs (Figure S5, Supporting Information), a calibration curve for fluorescence detection of influenza A (H4N6) virus was obtained using the standard dilution method (Figure S6A, Supporting Information). The linear range of the influenza A (H4N6) virus detection in complex media was 128–0.0012 hemagglutinin unit (HAU)/50 μL; the LOD was 0.00403 HAU/50 μL calculated using standard deviation method. Selectivity of the proposed fluorescence‐based sensor was performed on a series of similar viruses, confirming that the proposed assay is highly specific for the target analyte (Figure S6B, Supporting Information). Furthermore, the virus culture sample, that is, avian influenza A (H4N6), detection using proposed assay was validated with the commercial avian influenza A (H4N6) detection kit (Table S3, Supporting Information). Results indicated that the proposed fluorescence‐based sensor was superior over the commercial kit in terms of sensitivity. Thus, the proposed fluorescence biosensor enables the utilization of this technique in real‐life applications.

While examining the chiral responses for avian influenza A (H4N6) virus detection in complex media, results showed a linear response in the range of 128–0.0128 HAU/50 μL with LOD value of 0.0381 HAU/50 μL (Figure S7A, Supporting Information). Selectivity of the chiral‐based bioassay was also investigated using several similar types of viruses, and results confirmed that the proposed chirosensor is highly selective to the target analyte in complex media (Figure S7B, Supporting Information).

In comparison to the developed fluorescence‐based sensing mechanism, the chirosensor demonstrated less sensitivity. The bioanalytical potential of optical biosensors such as an exciton–plasmon interaction‐based optical biosensor, a surface‐enhanced Raman scattering (SERS)‐based biosensor, and a plasmonic biosensor—the interparticles distance is very crucial to obtain optical enhancement,^44^, ^45^ create “hot spots” and electric field enhancements, which ultimately limits the application of these techniques in the detection of relatively larger analytes, that is, different kinds of bacterium. To overcome these limitations, a chiral‐based biosensor would be a promising candidate because of its less dependency on interparticles distance. This exceptional capability of a chiroptical‐based sensor makes it easily distinguishable from other techniques, providing a viable means of detecting analytes with a wide range of diameters.^46^, ^47^

## 3 Conclusions

A bottom‐up synthetic route of blue‐emissive chiral MoS_2_ QDs has been introduced with the assistance of an autoclave. A series of characterizations was conducted using HRTEM, fluorescence spectrometry, circular dichroism spectroscopy, Raman spectroscopy, elementary analysis, UV–visible spectroscopy, and fluorescence lifetime measurement. As‐prepared MoS_2_ QDs were uniform in size (2.5 nm) and spherically shaped with distinct optical properties (i.e., both fluorescence and chiral). A dual‐mode optical sensor was subsequently developed using as‐prepared MoS_2_ QDs, and the results were validated using a conventional ELISA method and commercial kit. Furthermore, the practicability of the proposed dual‐mode optical sensor was investigated for viral culture detection in blood media. Results revealed that the developed sensor yielded a higher sensitivity than the conventional ELISA methods and commercial kits. Herein, we have demonstrated the optical‐based detection applications of MoS_2_ QDs; however, synthesized QDs would be suitable for other biosensing and bioimaging applications as well as optical‐based device applications.

## 4 Experimental Section

*Materials and Reagents*: Molybdenum (IV) sulfide (95 nm in diameter), poly‐l‐lysine (PLL), hydrogen peroxide (H_2_O_2_), and Nunc‐Immuno 96‐well plates were purchased from Sigma‐Aldrich (St. Louis, MO). l(+)‐ascorbic acid was purchased from Wako Pure Chemical Industries, Ltd. (Osaka, Japan). The anti‐influenza A (H5N1) virus hemagglutinin (HA) antibody [2B7] (ab135382, lot: GR100708‐16), recombinant influenza virus A (Avian/Vietnam/1203/04) (H5N1) (lot: GR301823‐1), goat anti‐mouse IgG, horseradish peroxidase (HRP)‐conjugated secondary antibody (Ab 97023, lot: GR 250300‐11), and immunoassay blocking buffer (Ab 171534, lot: GR 223418‐1) were purchased from Abcam, Inc. (Cambridge, UK). Recombinant influenza virus A (H1N1) (California) (CLIHA014‐2; lot: 813PH1N1CA) was brought from Cedarlane (Ontario, Canada). Influenza A (H5N2) hemagglutinin antibodies (anti‐H3N2 antibodies HA MAb, Lot: HB05AP2609), Influenza A (H7N9) hemagglutinin antibodies (Anti‐H7N9 antibody HA MAb, Lot: HB05JA1903), recombinant influenza virus A (H5N2) HA1 (A/Ostrich/South Africa/A/109/2006) (lot: LC09AP1021), recombinant influenza virus A (H7N8) HA1 (A/Mallard/Netherlands/33/2006) (lot: LC09AP1323), and recombinant influenza virus A (H7N9) HA1 (A/Shanghai/1/2013) (lot: LC09JA2702) were purchased from Sino Biological, Inc. (Beijing, China). Avian influenza H5N1 neuraminidase polyclonal antibody (Cat. PA5‐34949) was received from Invitrogen (Ontario, Canada). Anti‐H4 (A/environment/Maryland/1101/06) (H4N6) polyclonal antibody was purchased from MyBioSource Inc., San Diego, CA. Chicken whole blood (Cat. No. IR1‐080N) was received from Innovation Research, Michigan, USA. All experiments were performed using highly pure deionized (DI) water (>18 MΩ cm).

*Synthesis of MoS_2_ QDs*: MoS_2_ QDs were synthesized with the assistance of Benchtop Autoclave (Model: STE‐16‐C, China). Briefly, 1 mL (0.1 m) of l(+)‐ascorbic acid and MoS_2_ nanopowder (0.5 mg mL^−1^, 20 mL) were mixed in aqueous media and kept in an autoclave for 1 h at 120 °C. QDs samples were subsequently cooled at room temperature and the as‐prepared MoS_2_ QDs solution was yellow in color, whereas its bulk MoS_2_ nanopowder was dark gray. Here, l(+)‐ascorbic acid acts as a reducing agent, as a stabilizer, and also as a chiral ligand.

*Synthesis of Magnetic Nanoparticles (MNPs)*: Water‐soluble MNPs were prepared as previously reported.^48^ Shortly thereafter, FeCl_3_ (0.65 g, 4.0 mmol) and trisodium citrate (0.20 g, 0.68 mmol) were mixed in ethylene glycol (20 mL). Then, sodium acetate (1.20 g) was added in while stirring for 30 min. The mixture was sealed in a Teflon‐lined stainless steel autoclave, which was heated at 200 °C for 10 h. The black color end products of MNPs were then cooled down, followed by washing with ethanol and deionized water.

*Avian Influenza A (H4N6) Virus*: Influenza virus A (H4N6) (Avian influenza virus A/Duck/Czech/56 H4N6) was cultured in embryonated chicken eggs (11 d old) by inoculation into the allantoic cavity, as previously reported.^49^ Influenza virus HA protein was confirmed by hemagglutination assay. The total protein was measured using a BCA assay (Thermo Scientific) as per the provided manufacturer's protocol at a 50% tissue culture infective dose of 128 HAU/50 μL.

*Specificity of Antibodies toward Target Influenza A (H5N1) Virus*: A conventional ELISA method was conducted to check the selectivity of anti‐H5N1 HA and anti‐H5N1 NA antibodies toward avian influenza virus A/Vietnam 1203/04/2009 (H5N1). A total of 50 μL (1 μg mL^−1^) of virus solution in a PBS buffer solution (pH 7.5) was added to a PS plate and kept overnight at 4 °C. The next day, wells were rinsed three times with PBS buffer solution (pH 7.5), and an immunoassay blocking buffer (Ab 171534, 100 μL) was added and kept at room temperature for 2 h. After washing three times with PBS buffer solution (pH 7.5), anti‐H5N1 HA Ab (1 μg mL^−1^), anti‐H5N1 NA antibody (1 μg mL^−1^), anti‐H7N9 HA antibody (1 μg mL^−1^), and anti‐H5N2 HA antibody (1 μg mL^−1^), were subsequently added to each of the wells separately, and incubated for 1 h. Again, the wells were washed, and horseradish peroxidase labeled secondary antibody (50 μL, 1 μg mL^−1^) was added and incubated at room temperature for 1 h. Then, unbound or loosely bound secondary antibodies were washed out using PBS buffer (pH 7.5), and TMBZ (10 × 10^−9^ m)/H_2_O_2_ (5 × 10^−9^ m) solution was added to each well (50 μL) for 10 min at room temperature. The enzymatic reactions were stopped by adding 10% H_2_SO_4_ solution (50 μL/well), and the absorbance of the solutions was measured using a microplate reader (Cytation 5, BioTek Instruments Inc., Ontario, Canada) at 450 nm.

*Anti‐H5N1 HA (Ab 135382) Conjugation with MoS_2_ QDs*: Target virus‐specific antibodies (anti‐H5N1 HA Ab 135382) were bound to MoS_2_ QDs through electrostatic bonds. Briefly, 200 μL of positively charged PLL (0.1% wt, Sigma‐Aldrich) was mixed with 800 μL of negatively changed QDs (−19.03 eV) by gently stirring for 1 h at room temperature. At this stage, QDs were electrostatically covered by PLL, and was subsequently used to physically immobilize the anti‐H5N1 HA (Ab 135382) (1 μg mL^−1^ final concentration of antibody) on the surface of QDs. The mixture was then centrifuged (15 000 rpm for 10 min) and washed three times with PBS buffer to remove the unbound components. To check the binding between antibody and QDs, samples were blocked with 2% BSA (100 μL) for 2 h at room temperature. After the centrifugation and washing steps, 1 ng mL^−1^ (50 μL) of anti‐mouse IgG‐horseradish peroxidase (HRP) (Santa Cruz Biotechnology, CA) was added to each well, followed by incubation at room temperature for 1 h. Finally, a total of 100 μL TMBZ substrate solution (10 μg mL^−1^, TMBZ, 10% H_2_O_2_ in 100 × 10^−3^ m NaOAc, pH 6.0) was added to each well for 5–30 min at 25 °C. An enzymatic color (blue color) developed at this stage, and the reaction was stopped by adding 10% H_2_SO_4_ (100 μL). Additionally, absorbance was measured using a microplate reader.

*Binding of Anti‐H5N1 NA with Carboxyl‐Capped QDs Using ELISA*: Carboxyl‐capped MNPs were bound with amino groups of anti‐NA H5N1 antibodies through an amide bond. Shortly thereafter, 1 mL of MNPs was placed in a 1.5 mL microfuge tube, followed by the addition of 4 × 10^−3^ m of EDC, 10 × 10^−3^ m of NHS, and 1 μL anti‐H5N1 NA antibodies (5 ng mL^−1^), and the mixture was gently stirred at 10 °C. Unconjugated antibodies were separated through the use of external magnets, and washed three times before further use.

*Validation Study of Proposed Method*: The validation of the proposed sensing method was conducted with a commercially available ELISA kit for avian influenza A H5N1 (Cat. No. MBS9324259, MyBioSource, Inc., San Diego, CA). Several concentrated viral solutions were made using the sample diluent included in the commercial ELISA kit, strictly adhering to the manufacturer's protocol during bioassay. The color developed with different levels of absorbance intensity related to the viral concentration, and was recorded using a microplate reader. Influenza A (H4N6) virus detection was also validated with another commercially available ELISA kit (Cat. No. NS‐E10156, Novatein Biosciences, Woburn, MA). The assay procedure was strictly followed as mentioned in the protocol booklet.

*Spectroscopy and Structural Characterization*: TEM images were taken using Tecnai TEM (FEI Tecnai G2 F20, Ontario, Canada). Zetasizer Nano ZS (Malvern Instruments Ltd., Worcestershire, UK) was used for surface charge measurements. A circular dichroism spectrum was recorded using JASCO CD Spectrometer (Model: J‐815, Easton, USA). Magnetic properties of MNPs were measured using SQUID magnetometry at 300 K (Quantum Design MPMS‐7 SQUID, San Diego, CA). The binding of antibodies and MNPs was characterized by FTIR spectroscopy (FT/IR6300, JASCO Corp., Tokyo, Japan). Scanning electron microscope images were acquired by Quanta FEG 250 (Oregon, USA).

The limit of detection (LOD) was calculated based on the standard deviation method as reported previously^50^, ^51^, ^52^, ^53^

(4) $$\mathrm{LOD}\;\geq \;e^{\frac{3.3{\mathrm{SD}}_{\mathrm{blank}}}{b}}$$

In Equation (4), SD_blank_ and *b* indicate the standard deviation of the mean blank signal and the slope of the linear regression curve, respectively. All experiments were performed in triplicate and repeated three times with similar results. Bars display mean ± s.d., and statistical analysis was performed using Student's *t*‐test.

## Conflict of Interest

The authors declare no conflict of interest.

## Supporting information

Supplementary
