# Supplementary material for: Chiral MoS2 Quantum Dots: Dual‐Mode Detection Approaches for Avian Influenza Viruses
Source: Glob Chall. 2018 Mar 12;2(4):1700071. doi: 10.1002/gch2.201700071 (PMC6607333; doi:10.1002/gch2.201700071)
Supplement: Supplementary file 1 — Supplementary [file GCH2-2-1700071-s001.pdf]

Copyright WILEY-VCH Verlag GmbH & Co. KGaA, 69469 Weinheim, Germany, 2018.

Global Challenges
Open Access

Supporting Information

for *Global Challenges,* DOI: 10.1002/gch2.201700071

Chiral MoS_2 Quantum Dots: Dual-Mode Detection Approaches for Avian Influenza Viruses

*Syed Rahin Ahmed and Suresh Neethirajan**

Supporting Information

Chiral MoS_2 Quantum Dots: Dual-Mode Detection Approaches for Avian Influenza Viruses using Magnetochirofluorescent Nanohybrids

Syed Rahin Ahmed[1] and Suresh Neethirajan[1*]

[1]BioNano Laboratory, School of Engineering, University of Guelph, Guelph, Ontario, Canada N1G 2W1

[*]Corresponding author: sneethir@uoguelph.ca (SN)

Confirmation of antibodies specificity towards target virus

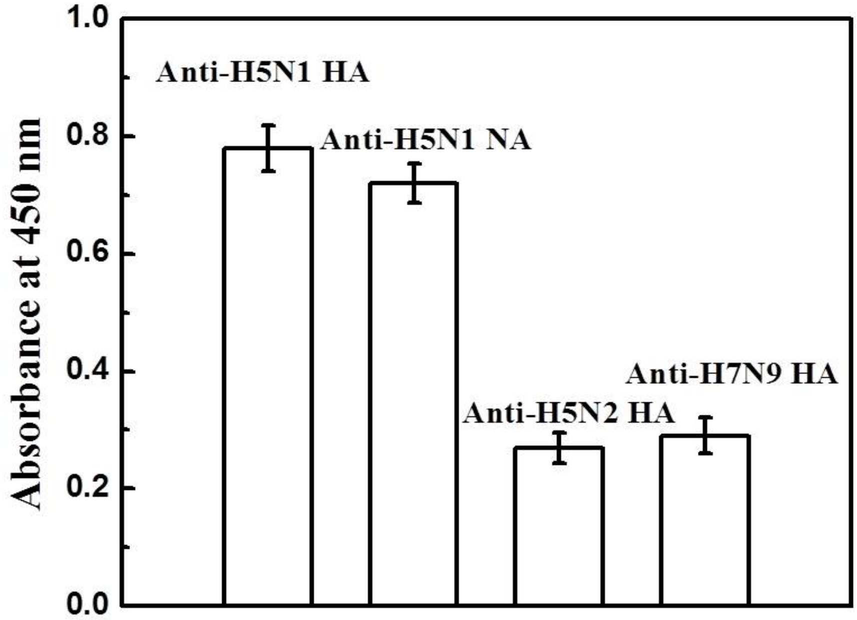

Figure S1: ELISA results of antibodies specificity towards target viruses.

Binding confirmation of anti-H5N1 HA Ab 135382 with QDs and anti-H5N1 NA Ab with MNPs

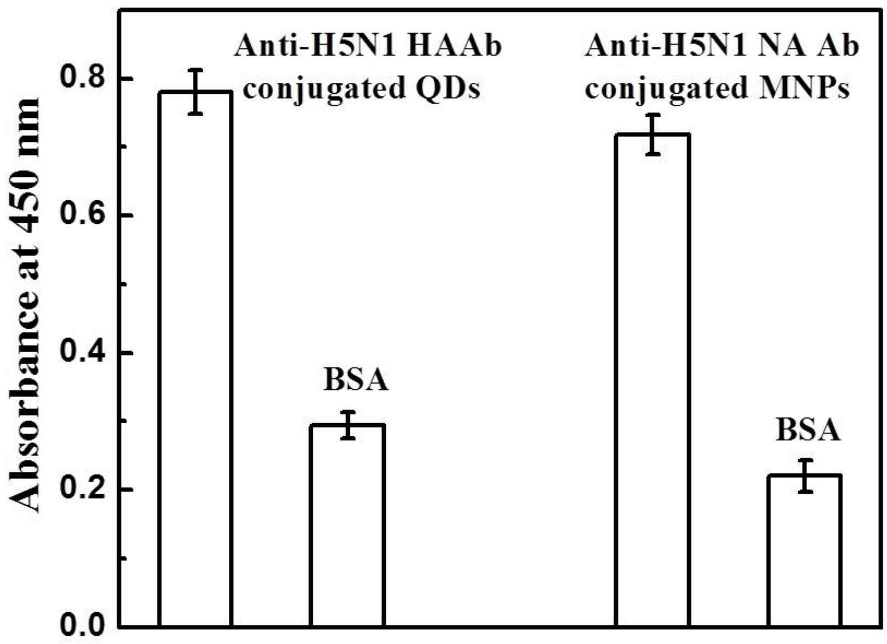

Figure S2: ELISA results of antibodies binding with MoS_2 QDs and MNPs.

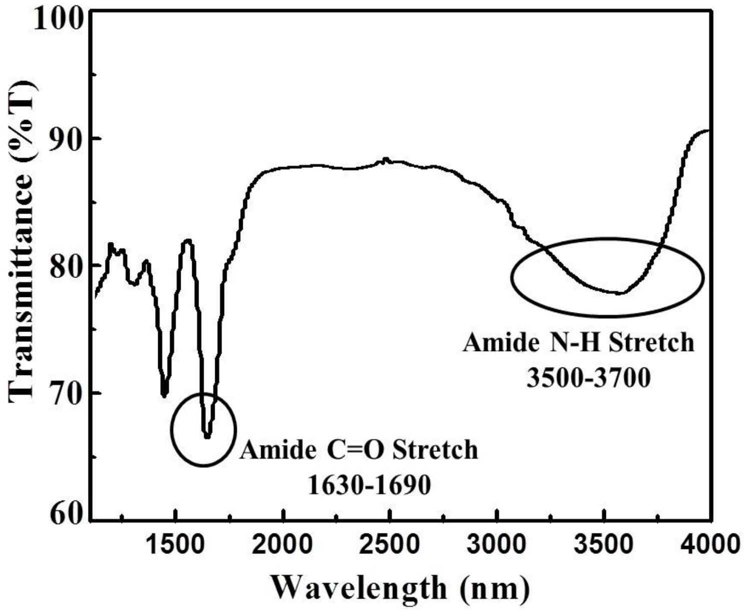

Figure S3: FTIR spectra of antibodies conjugated MNPs.

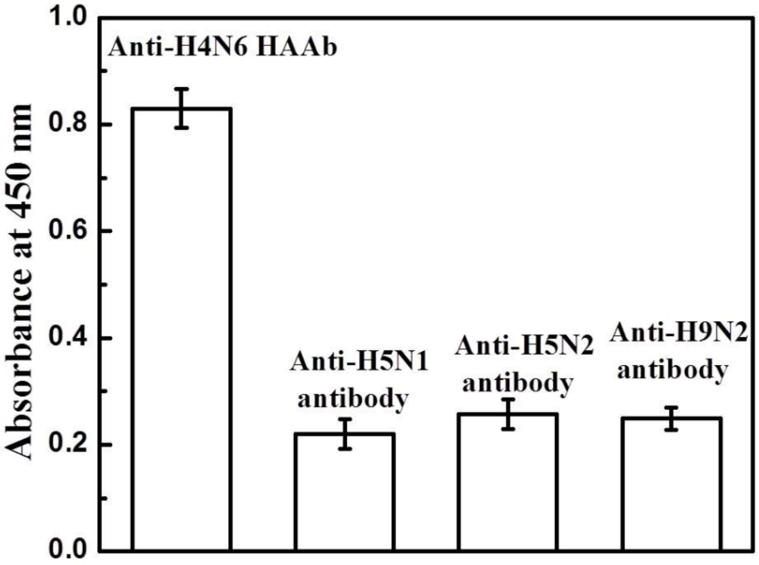

Figure S4: ELISA results of antibodies specificity towards avian influenza A (H4N6) virus.

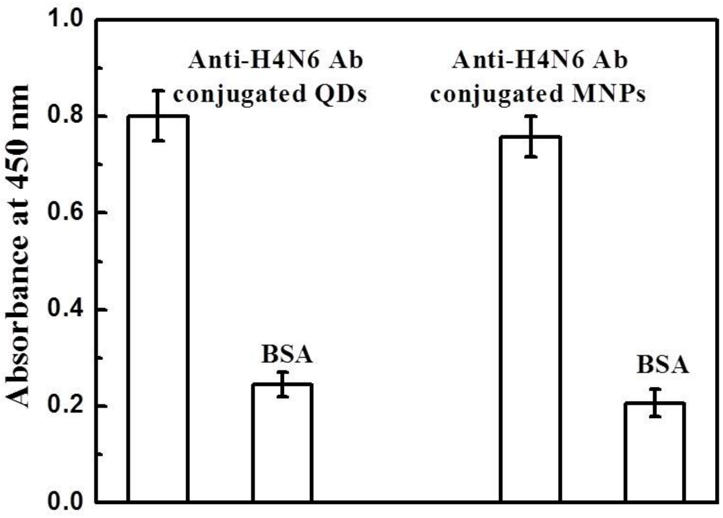

Figure S5: Binding confirmation of antibodies with MoS_2 QDs and MNPs through ELISA method.

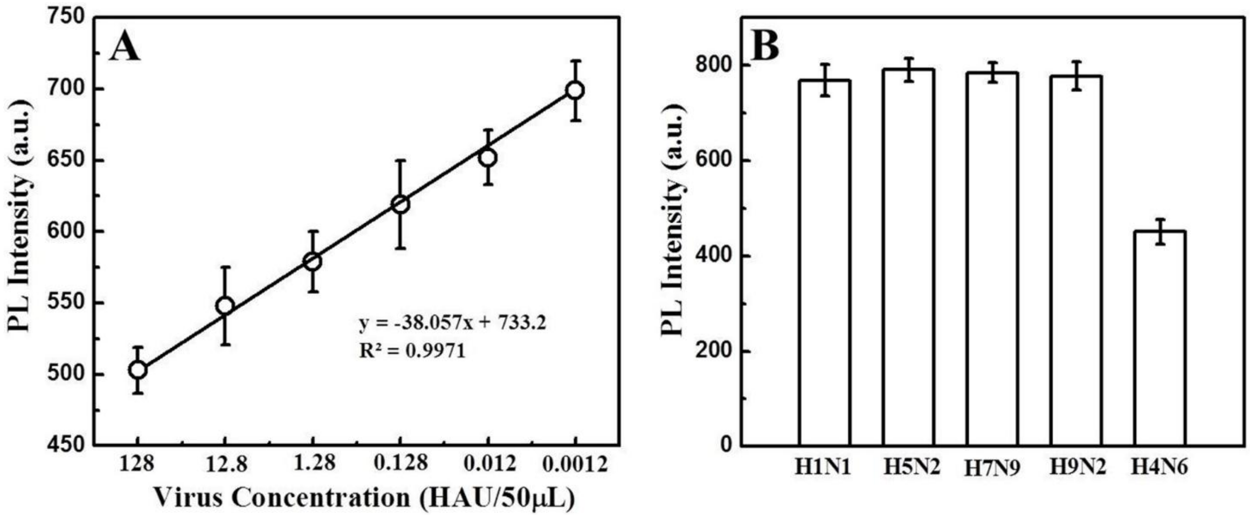

Figure S6: Fluorescence detection of virus culture sample: (A) calibration curve of avian influenza virus A (H4N6) concentration VS fluorescence responses; and (B) selectivity results of detection in chicken blood media.

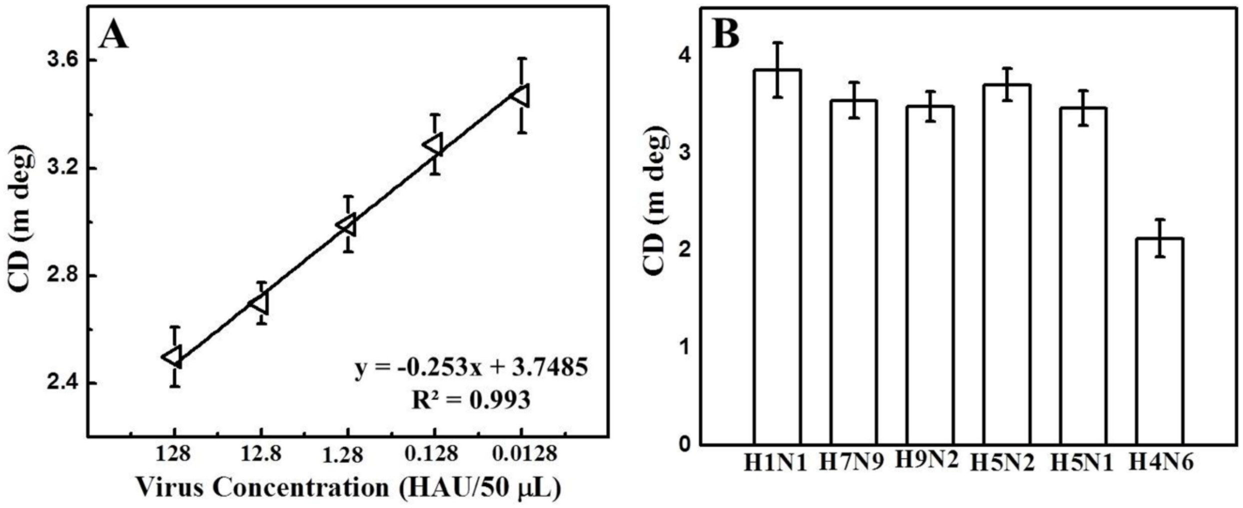

Figure S7: Chiral-based optical detection of virus culture sample: (A) calibration curve of avian influenza virus A (H4N6) concentration VS chiral responses; and (B) selectivity results of chiral-based detection in chicken blood media.

Table S1: Step by step analysis of zeta potential value

	Zeta potential (mV)		
	MoS_2 QDs	after conjugation with Poly-l lysine (PLL)	after conjugation with antibodies
MoS_2 QDs	-20.52	+10.31	-3.83

Table S2: Comparison of avian influenza virus A (Avian/Vietnam/1203/04) (H5N1) detection using different methods

Detection method	Virus concentration (pg/mL)								
	10^7	10^6	10^5	10^4	10^3	10^2	10	1	0
Proposed PL sensor	+	+	+	+	+	+	+	–	–
Proposed CD sensor	+	+	+	+	+	+	–	–	–
Conventional ELISA	+	+	+	+	+	–	–	–	–
Commercial kit	+	+	+	+	+	–	–	–	–

Table S3: Comparison study of avian influenza A (H4H6) virus detection

Detection method	Virus concentration (HAU/ 50 µL)						
	128	12.8	1.28	0.128	0.0128	0.00128	0.000128
Proposed PL sensor	+	+	+	+	+	+	–
Proposed CD sensor	+	+	+	+	+	–	–
Commercial kit	+	+	+	+	–	–	–
